# On the Helmert-blocking technique: its acceleration by block Choleski decomposition and formulae to insert observations into an adjusted network

**DOI:** 10.1098/rsos.140417

**Published:** 2015-04-22

**Authors:** Eduardo Del Rio, Leonardo Oliveira

**Affiliations:** 1 Geodesy Laboratory, Department of Geophysics, Graduate School of Science, Kyoto University, 1/252 Kyoto, Japan; 2 Instituto Militar de Engenharia, Programa de Pós-graduação em Engenharia de Defesa, Seção de Engenharia Cartográfica, SE/6, Rio de Janeiro, Brazil; 3 Pontifícia Universidade Católica do Rio de Janeiro, Programa de Pós-graduação em Metrologia para Qualidade e Inovação, Rio de Janeiro, Brazil

**Keywords:** large geodetic networks, block system, Helmert-blocking, block Choleski decomposition, insertion of new observations

## Abstract

The Helmert-blocking technique is a common approach to adjust large geodetic networks like Europeans and Brazilians. The technique is based upon a division of the network into partial networks called blocks. This way, the global network adjustment can be done by manipulating these blocks. Here we show alternatives to solve the block system that arises from the application of the technique. We show an alternative that optimizes its implementation as the elapsed processing time is decreased by about 33%. We also show that to insert observations into an adjusted network it is not necessary to readjust the whole network. We show the formulae to insert new observations into an adjusted network that are more efficient than simply readjusting the whole new network.

## 2. Introduction

### 2.1 Review of the Helmert-blocking technique

This work deals with the Helmert-blocking (HB) technique, an approach designed for the adjustment of large geodetic networks, like Europeans and Brazilians. Developed in the nineteenth century by the geodesist Helmert [1], some recent examples of the use of the HB technique are as follows: to adjust the North American Datum of 1983 (NAD83) and to integrate the Canadian geodetic network to NAD83 [2], where Canada developed for this purpose, in the 1980s, the computer system GHOST (Geodetic Adjustment using Helmert Blocking of Space and Terrestrial Data) [3]. This software tool had been used in Brazil since the 1990s by the Institute for Geography and Statistics. The transition to GHOST in Brazil occurred when computer systems based on classical methods of adjustment started to become unfeasible [4].

GHOST was also used in Brazil to adjust the horizontal network of the Geodetic Brazilian network (RGB) to SIRGAS2000, and it is also being employed in the adjustment of the levelling network of RGB to SIRGAS2000 [4–6]. Furthermore, the HB technique has also been recently used in other parts of the world. The International Association of Geodesy Reference Frame Sub-Commission for Europe used the HB technique in the adjustment of a network of continuous operating GPS stations (EPN) [7]. Nocquet *et al.* [8] also used the HB technique on the generation of a plate kinematics model for Nubia–Somalia region.

The HB technique consists in subdividing the global geodetic network into partial networks referred to as blocks. This way, the adjustment of the global network can be done through the manipulation of these smaller blocks. Regarding the division of the global geodetic network into blocks, several criteria may be used [1,2], but this issue is out of the scope of this article.

### 2.2 Outline of the paper

HB was and is still currently used on the adjustment of geodetic networks. Recent uses of HB include other kinds of data as well (e.g. GPS and plate kinematics). Thus, considering the small number of references to the mathematical formulae of this technique and considering that the deductions are taken for granted or simplified in these instances, here we formally deduce the formulae of the technique in detail but concisely. In contrast to Wolf [1], here we derive HB general formulae from least squares (LS) formula based on properties of block matrices (§3). Then we derive formulae to insert observations in a geodetic network adjusted in advance based on this *a priori* adjusted solution (§4). Of note, the derivation in §3 serves as a support for the proposition of the formulae for the insertion of observations.

In previous work, we, together with other colleagues, developed a framework to deduce and implement the technique under Matlab [9]. The proposed framework led us to derive alternatives to implement the HB technique and to conceive softwares for the adjustment of geodetic networks based both on the classical HB approach [1] and on our alternatives which are: HB by block Choleski decomposition (HBC) and HB by block Gaussian elimination (HBG). However, the tests developed and presented by Lema *et al*. [9] were very restricted due to the nature of the generation of random matrices and especially the small size of the junction unknowns vector (hereafter referred to as junction vector). Here we take the simulations to another level and we present broader tests that validate the deductions and implementation and show once more that the proposed modification in the technique optimizes it and that this optimization is enhanced when the junction vector is very large (§5), which is the case for large geodetic networks.

Previously, in [9], we verified that the proposed changes optimized the implementation but without quantifying this improvement (due to the restricted data generated in this previous simulation). So, here, we extensively tested the technique under several geodetic networks of varying configurations.

## 3. Deduction of the Helmert-blocking's formulae

While handling observations of large geodetic networks, it is inevitable to face thousands or even millions of equations involving thousands of variables. For instance, Schwarz & Wade [2] handled 1 785 772 observations and 928 735 unknowns in the adjustment of NAD83, in which Wolf's [1] formulation of the HB technique was adopted. As a consequence, it is inviable to employ the classic LS method to the adjustment of the geodetic data of the whole network due to the great dimension of the matrices involved in it.

On the other hand, in many instances, these equations are previously organized in observation sets called blocks (or can be organized as such), giving particular characteristics to this system of equations [1,2]. Such block configuration is more adequate to exploit the sparseness of the network's matrix. And the HB technique manipulates these blocks to give an adjusted solution to the global network while taking advantage of this sparseness of the network's matrix [2].

We will include here the latest derivation of HB's formulae which goes after [9]—we include these derivations here because they will be important for §4 and because [9] is in Portuguese only.

The variables of each block may be classified according to their participation in neighbouring blocks.

— *Junction unknowns.* These are the variables corresponding to stations belonging to more than one block of the geodetic network.
— *Interior unknowns.* These are the variables belonging to only a single block of the geodetic network.

Assume the dataset of a given geodetic network is divided in *n* blocks, each having at least one benchmark, all benchmarks referring to the same reference system. Assuming that the network as a whole has at least one junction unknown (the adjustment in the absence of junction unknowns is as outlined in §4.5 which means that each block can be adjusted individually as they do not have any influence on the adjusted solution of the other blocks) and that all the observations have the same quality (the solution considering individual weights for the observations is analogous and will be outlined later, in §3.2), we have, for a given block *i* of this network [1]:

3.1 $$A_{i}x_{i}+B_{i}y-l_{i}=v_{i},\;\forall \;i,\;1\leq i\leq n,$$

where *x*_*i*_=vector corresponding to the interior unknowns of block *i*; *y*=vector corresponding to the junction unknowns; *A*_*i*_=matrix of the interior unknowns of block *i*; *B*_*i*_=matrix of the junction unknowns of block *i*; *l*_*i*_=vector of the observations corresponding to block *i*; *v*_*i*_=vector of the residuals of block *i*'s observation.

**Figure 1.**
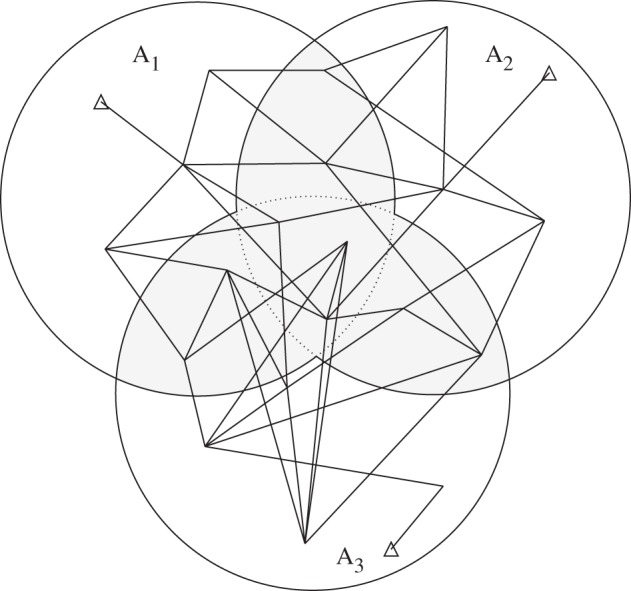
Levelling network composed of three blocks that we used in a previous simulation of the HB technique [9]. Here, this network is used as a seed that generates much larger networks. Each block is uniquely matched to the interior of a circle. The stations belonging to the shaded region are the ones comprising junction unknowns. The other stations correspond to the interior unknowns. Triangles represent benchmarks, i.e. points with known coordinates.

Figure 1 shows the defined terms, some of which are involved in equation (3.1). In this figure can be observed a geodetic network partitioned in three blocks, its junction stations, its interior stations and the measured height differences. For further examples of the block's division and especially the different levels of junction unknowns (this concerns its division into subvectors, see §3.1) we suggest the reader to refer to [2,10].

Rewriting equation (3.1), an alternative representation through block matrices of the global geodetic network *Ax*−*l*=*v* follows:

3.2 $$\left(\begin{array}{ccccc} A_{1} & & & & B_{1}\\ & A_{2} & & & B_{2}\\ & & \ddots & & \vdots \\ & & & A_{n} & B_{n} \end{array}\right)\left(\begin{array}{c} x_{1}\\ x_{2}\\ \vdots \\ x_{n}\\ y \end{array}\right)-\left(\begin{array}{c} l_{1}\\ l_{2}\\ \vdots \\ l_{n} \end{array}\right)=\left(\begin{array}{c} v_{1}\\ v_{2}\\ \vdots \\ v_{n} \end{array}\right).$$

Applying LS method to the global geodetic network provides an adjusted solution *x* given by *A*^*t*^*Ax*=*A*^*t*^*l* [11]. Equation (3.2) will give rise to the corresponding block representations of *A*^*t*^*A* and *A*^*t*^*l*.

3.3 $$A=\left(\begin{array}{ccccc} A_{1} & & & & B_{1}\\ & A_{2} & & & B_{2}\\ & & \ddots & & \vdots \\ & & & A_{n} & B_{n} \end{array}\right)⟺A^{t}=\left(\begin{array}{cccc} A_{1}^{t} & & & \\ & A_{2}^{t} & & \\ & & \ddots & \\ & & & A_{n}^{t}\\ B_{1}^{t} & B_{2}^{t} & \text{…} & B_{n}^{t} \end{array}\right).$$

The HB technique corresponds to the LS method applied not to *Ax*−*l*=*v* but to its block representation given by equation (3.2). Thus, from equations (3.2) and (3.3) the block representations for *A*^*t*^*A* and *A*^*t*^*l* follow:

3.4 $$A^{t}A=\left(\begin{array}{ccccc} A_{1}^{t}A_{1} & & & & A_{1}^{t}B_{1}\\ & A_{2}^{t}A_{2} & & & A_{2}^{t}B_{2}\\ & & \ddots & & \vdots \\ & & & A_{n}^{t}A_{n} & A_{n}^{t}B_{n}\\ B_{1}^{t}A_{1} & B_{2}^{t}A_{2} & \text{…} & B_{n}^{t}A_{n} & \sum_{1\leq k\leq n}B_{k}^{t}B_{k} \end{array}\right)$$

and

3.5 $$A^{t}l=\left(\begin{array}{c} A_{1}^{t}l_{1}\\ A_{2}^{t}l_{2}\\ \vdots \\ A_{n}^{t}l_{n}\\ \sum_{1\leq k\leq n}B_{k}^{t}l_{k} \end{array}\right).$$

The solution by the HB technique is achieved by solving the matrix equation *A*^*t*^*Ax*=*A*^*t*^*l*, where *x*=(*x*_1_*x*_2_⋯*x*_*n*_*y*)^T^. Hence, it is identical to the solution of LS when applied directly to the global geodetic network. Yet, further algebraic manipulations will yield the final HB expression.

Equations (3.4) and (3.5) yield

3.6 $$\left(\begin{array}{c} A_{1}^{t}A_{1}x_{1}+A_{1}^{t}B_{1}y\\ A_{2}^{t}A_{1}x_{2}+A_{2}^{t}B_{2}y\\ \vdots \\ A_{n}^{t}A_{n}x_{n}+A_{n}^{t}B_{n}y\\ \sum_{1\leq k\leq n}(B_{k}^{t}A_{k}x_{k}+B_{k}^{t}B_{k}y) \end{array}\right)=\left(\begin{array}{c} A_{1}^{t}l_{1}\\ A_{2}^{t}l_{2}\\ \vdots \\ A_{n}^{t}l_{n}\\ \sum_{1\leq k\leq n}B_{k}^{t}l_{k} \end{array}\right).$$

Making the following substitutions:

3.7 $$N_{i}=A_{i}^{t}A_{i};\;R_{i}=A_{i}^{t}B_{i};\;M_{i}=B_{i}^{t}B_{i};\;u_{i}=A_{i}^{t}l_{i};\;w_{i}=B_{i}^{t}l_{i},$$

equation (3.6) is expressed in a shorter expression:

3.8 $$\left.\begin{array}{rl} & N_{i}x_{i}+R_{i}y=u_{i},\;\forall i,\;1\leq i\leq n\\ \;\text{and}\;\; & \sum_{1\leq k\leq n}(R_{k}^{t}x_{k}+M_{k}y)=\sum_{1\leq k\leq n}w_{k}. \end{array}\right\}$$

Isolating *x*_*i*_ in the first line of equation (3.8) and substituting it on the second line of equation (3.8), the HB solution of the geodetic network arises

3.9 $$\left.\begin{array}{rl} & \sum_{1\leq k\leq n}(M_{k}+R_{k}^{t}N_{k}^{-1}R_{k})y=\sum_{1\leq k\leq n}(w_{k}-R_{k}^{t}N_{k}^{-1}u_{k})\\ \;\text{and}\;\; & x_{i}=N_{i}^{-1}(u_{i}-R_{i}y),\;\forall i,\;1\leq i\leq n. \end{array}\right\}$$

The network's adjusted solution is obtained by calculating primarily *y* from the first line of equation (3.9). Then, each *x*_*i*_ is determined by substituting this computed *y*-value onto the second line of equation (3.9). Usually, the Choleski method is to be applied to solve the system in the junction vector *y* [1]. After all, $\sum_{1\leq k\leq n}(M_{k}+R_{k}^{t}N_{k}^{-1}R_{k})$ is a symmetric matrix and weighted in its diagonal, a feature of symmetric positive definite matrices [11]. This precludes the need for pivoting and allows a fast Choleski algorithm to solve the system.

### 3.1 Junction unknowns represented by subvectors

In most cases (as for large geodetic networks), the great dimension of the junction vector, together with particular properties of the geodetic network in consideration, makes it expedient to divide it in subvectors [1]. For instance, NAD83 adjustment had a sizable junction vector for each of its blocks, generally comparable to the blocks' interior vector [2]. In such a case, the junction vector, *y*, grows too large and this becomes a hindrance to its direct manipulation. Without alternative approaches, the junction vector would extend to hundreds of thousands of unknowns, and thus its corresponding system would have equations to this same amount and the initial division of the network into blocks would become meaningless. Meanwhile, despite the network's block division, the blocks *B*_*i*_ are still sparse, as actually, very few, or no junction unknown, belongs to all blocks. So, here we will show how to achieve an adjusted solution starting from a division of the junction vector into subvectors:

3.10 $$y=\left(\begin{array}{c} y_{1}\\ y_{2}\\ \vdots \\ y_{d} \end{array}\right).$$

This for instance, yields the following substitutions:

3.11 $$\begin{array}{rl} B_{i} & =\left(\begin{array}{cccc} B_{1i} & B_{2i} & \ldots & B_{di} \end{array}\right), \end{array}$$

3.12 $$\begin{array}{rl} R_{i} & =\left(\begin{array}{cccc} A_{i}^{t}B_{1i} & A_{i}^{t}B_{2i} & \ldots & A_{i}^{t}B_{di} \end{array}\right)=\left(\begin{array}{cccc} R_{1i} & R_{2i} & \ldots & R_{di} \end{array}\right), \end{array}$$

3.13 $$\begin{array}{rl} M_{i} & =B_{i}^{t}B_{i}=\left(\begin{array}{c} cB_{1i}^{t}\\ B_{2i}^{t}\\ \vdots \\ B_{di}^{t} \end{array}\right)\left(\begin{array}{cccc} B_{1i} & B_{2i} & \ldots & B_{di} \end{array}\right)\\ & \;\Rightarrow M_{i}=\left(\begin{array}{cccc} B_{1i}^{t}B_{1i} & B_{1i}^{t}B_{2i} & \ldots & B_{1i}^{t}B_{di}\\ B_{2i}^{t}B_{1i} & B_{2i}^{t}B_{2i} & \ldots & B_{2i}^{t}B_{di}\\ \vdots & \vdots & \ddots & \vdots \\ B_{di}^{t}B_{1i} & B_{di}^{t}B_{2i} & \ldots & B_{di}^{t}B_{di} \end{array}\right)=\left(\begin{array}{cccc} M_{1i} & S_{12i} & \ldots & S_{1di}\\ S_{12i}^{t} & M_{2i} & \ldots & S_{2di}\\ \vdots & \vdots & \ddots & \vdots \\ S_{1di}^{t} & S_{2di}^{t} & \ldots & M_{di} \end{array}\right) \end{array}$$

3.14 $$\begin{array}{rl} \;\text{and}\;w_{i} & =\left(\begin{array}{c} B_{1i}^{t}\\ B_{2i}^{t}\\ \vdots \\ B_{di}^{t} \end{array}\right)l_{i}\Leftrightarrow w_{i}=\left(\begin{array}{c} B_{1i}^{t}l_{i}\\ B_{2i}^{t}l_{i}\\ \vdots \\ B_{di}^{t}l_{i} \end{array}\right)=\left(\begin{array}{c} w_{1i}^{t}\\ w_{2i}^{t}\\ \vdots \\ w_{di}^{t} \end{array}\right). \end{array}$$

The computation of the junction vector *y* is still done by equation (3.9). But, in this case the system to be solved is a block system given by equation (3.15), according to the representations adopted in equations (3.10)–(3.14):

3.15 $$\sum_{1\leq i\leq n}\left(\begin{array}{cccc} M_{1i}^{\prime } & S_{12i}^{\prime } & \ldots & S_{1di}^{\prime }\\ S_{12i}^{t}\prime & M_{2i}^{\prime } & \ldots & S_{2di}^{\prime }\\ \vdots & \vdots & \ddots & \vdots \\ S_{1di}^{t}\prime & S_{2di}^{t}\prime & \ldots & M_{di}^{\prime } \end{array}\right)\left(\begin{array}{c} y_{1}\\ y_{2}\\ \vdots \\ y_{d} \end{array}\right)=\sum_{1\leq i\leq n}\left(\begin{array}{c} w_{1i}^{\prime }\\ w_{2i}^{\prime }\\ \vdots \\ w_{di}^{\prime } \end{array}\right),$$

where

3.16 $$\left.\begin{array}{rlr} M_{ki}^{\prime } & =M_{ki}-R_{ki}^{t}N_{i}^{-1}R_{ki}, & \;k,\;1\leq k\leq d,\\ S_{kli}^{\prime } & =S_{kli}-R_{ki}^{t}N_{i}^{-1}R_{li}, & \;k,\;1\leq k<l\leq d\\ \;\text{and}\;\;w_{ki}^{\prime } & =w_{ki}-R_{ki}^{t}N_{i}^{-1}u_{i}, & \;k,\;1\leq k\leq d. \end{array}\right\}$$

An additional change of variables yields a concise expression

3.17 $$\left.\begin{array}{rl} \sum_{1\leq k\leq n}M_{ki}^{\prime } & =M_{k}*;\\ \sum_{1\leq k\leq n}S_{kli}^{\prime } & =S_{kl}*;\;\forall k,l,\;1\leq k<l\leq d\\ \;\text{and}\;\;\sum_{1\leq k\leq n}w_{ki}^{\prime } & =w_{k}*. \end{array}\right\}$$

The block system corresponding to the subvectors of the junction vector *y* is thus as follows:

3.18 $$\left(\begin{array}{cccc} M_{1i}* & S_{12i}* & \ldots & S_{1di}*\\ S_{12i}^{t}* & M_{2i}* & \ldots & S_{2di}*\\ \vdots & \vdots & \ddots & \vdots \\ S_{1di}^{t}* & S_{2di}^{t}* & \ldots & M_{di}* \end{array}\right)\left(\begin{array}{c} y_{1}\\ y_{2}\\ \vdots \\ y_{d} \end{array}\right)=\left(\begin{array}{c} w_{1i}*\\ w_{2i}*\\ \vdots \\ w_{di}* \end{array}\right).$$

To solve the block system of equation (3.18), one can turn to the literature, and use standardized approaches. The one presented by Wolf [1] resembles a Gauss–Jordan elimination adapted for block matrices; however, it is not formally presented. In fact, the term block system for this equation is not mentioned nor is the technique used (Gauss–Jordan elimination). Therefore, we consider the quality of Wolf's [1] approach inferior to a Gaussian elimination, i.e. a block Gaussian elimination [11], as a Gaussian elimination can be from 1.5 to 3 times more efficient than Gauss–Jordan elimination [12]. Furthermore, here we have formally divided the junction vector into subvectors, making a thorough deduction, as Wolf's [1] is concise.

As presented in the previous subsection, the matrix that makes up the system for the junction unknowns is symmetric and generally positive definite as its elements which are greater in absolute value are spread along its diagonal. So here, to take advantage of this characteristic, we also propose to use the Choleski block-triangular decomposition to solve this block system, since block Choleski's algorithm demands *n*^3^/3 floating point operations (flops), instead of 2*n*^3^/3 demanded by a block Gaussian algorithm [11]. In §5, we present a numerical experiment to attest the validity of such HBC approach. We evaluate its efficiency and accuracy, showing that as expected, it is more efficient than HBG while being just as accurate.

For details on how to implement HBC and HBG refer to appendix A. Another option yet unexplored in the HB context is the use of iterative methods, e.g. a block implementation of the conjugate gradient method might also be of help in future investigations.

### 3.2 Introduction of weights in the observations

In most cases, each observation has a weight corresponding to the quality of its measurement—e.g. the inverse of its variance (note that usually the covariance between observations is null). Let *P*_*i*_ be the weight matrix corresponding to the set of block *i*'s observations, which has as observations vector *l*_*i*_, equation (3.1). Therefore, the geodetic network as a whole has the following weight matrix:

3.19 $$P=\left(\begin{array}{cccc} P_{1} & & & \\ & P_{2} & & \\ & & \ddots & \\ & & & P_{n} \end{array}\right).$$

Taking *P* as the weight matrix for the geodetic network, the adjusted solution *x* by LS method becomes the solution to *A*^*t*^*PAx*=*A*^*t*^*Pl*. A way to find the adjusted solution *x* by the HB technique is to consider the equivalent system *A*^*t*^(*PA*)*x*=*A*^*t*^(*Pl*), where

3.20 $$PA=\left(\begin{array}{ccccc} P_{1}A_{1} & & & & P_{1}B_{1}\\ & P_{2}A_{2} & & & P_{2}B_{2}\\ & & \ddots & & \vdots \\ & & & P_{n}A_{n} & P_{n}B_{n} \end{array}\right)$$

and

3.21 $$Pl=\left(\begin{array}{c} P_{1}l_{1}\\ P_{2}l_{2}\\ \vdots \\ P_{n}l_{n} \end{array}\right).$$

Then, equations (3.20) and (3.21) may be achieved from equation (3.2) substituting each *A*_*i*_ for *P*_*i*_*A*_*i*_, each *B*_*i*_ for *P*_*i*_*B*_*i*_ and each *l*_*i*_ for *P*_*i*_*l*_*i*_. Similarly, to obtain the adjusted solution to the global geodetic network by the HB technique considering weighted observations, it suffices to make the corresponding changes of variables in the deduction already done. This way, the adjusted solution by the HB technique is also given by equations (3.7), (3.12)–(3.14), where the following changes of variables are to be made:

3.22 $$\begin{array}{rl} N_{i} & =A_{i}^{t}P_{i}A_{i};\;R_{i}=A_{i}^{t}P_{i}B_{i};\;M_{i}=B_{i}^{t}P_{i}B_{i};\;u_{i}=A_{i}^{t}P_{i}l_{i};\;w_{i}=B_{i}^{t}P_{i}l_{i}; \end{array}$$

3.23 $$\begin{array}{rl} R_{i} & =(A_{i}^{t}P_{i}B_{1i}\;A_{i}^{t}P_{i}B_{2i}\;\ldots \;A_{i}^{t}P_{i}B_{di})=(R_{1i}\;R_{2i}\;\ldots \;R_{di}) \end{array}$$

3.24 $$\begin{array}{rl} M_{i} & =\left(\begin{array}{cccc} B_{1i}^{t}P_{i}B_{1i} & B_{1i}^{t}P_{i}B_{2i} & \ldots & B_{1i}^{t}P_{i}B_{di}\\ B_{2i}^{t}P_{i}B_{1i} & B_{2i}^{t}P_{i}B_{2i} & \ldots & B_{2i}^{t}P_{i}B_{di}\\ \vdots & \vdots & \ddots & \vdots \\ B_{di}^{t}P_{i}B_{1i} & B_{di}^{t}P_{i}B_{2i} & \ldots & B_{di}^{t}P_{i}B_{di} \end{array}\right)=\left(\begin{array}{cccc} M_{1i} & S_{12i} & \ldots & S_{1di}\\ S_{12i}^{t} & M_{2i} & \ldots & S_{2di}\\ \vdots & \vdots & \ddots & \vdots \\ S_{1di}^{t} & S_{2di}^{t} & \ldots & M_{di} \end{array}\right) \end{array}$$

3.25 $$\begin{array}{rl} \;\text{and}\;w_{i} & =\left(\begin{array}{c} B_{1i}^{t}P_{i}l_{i}\\ B_{2i}^{t}P_{i}l_{i}\\ \vdots \\ B_{di}^{t}P_{i}l_{i} \end{array}\right)=\left(\begin{array}{c} w_{1i}^{t}\\ w_{2i}^{t}\\ \vdots \\ w_{di}^{t} \end{array}\right). \end{array}$$

## 4. Insertion of observations in an adjusted geodetic network

Now, given a network whose adjusted solution was already computed, the problem is to insert new blocks into it without readjusting the entire network. Based on the pre-determined solution, to compute the adjusted solution of the unknowns of the inserted blocks and the corrections to be added to the original pre-determined solution.

Concerning the blocks to be inserted, given one of them, there are four possibilities for the unknowns therein: first case, they comprise unknowns of the original network only; second case, they have their own interior unknowns but junction unknowns in common with the original network; third case, they have their own junction unknowns but interior unknowns in common with the original network; and fourth case, they have no unknown in common with the original network.

### 4.1 A general routine to insert new blocks in an adjusted network

A general routine to adjust the network based on the adjusted solution of the original one without reprocessing the entire network is as follows:

1. Organize the blocks to be inserted into four sets, a set for first case blocks, a set for second case blocks, a set for third case blocks and a set for fourth case blocks.
2. Compute corrections inserting only the first case blocks based on equations (4.2) and (4.3).
3. Insert the second case blocks and compute the adjusted solution for its unknowns and the second corrections based on equations (4.4) and (4.5) (second case blocks are inserted in the network comprising the original one plus first case blocks).
4. Insert the third case blocks and compute the adjusted solution for its unknowns and the third corrections based on equations (4.6) and (4.7) (third case blocks are inserted in the network comprising the original one plus first and second case blocks).
5. Compute the adjusted solution for the unknowns of the blocks enclosed in the fourth case separately using standard HB or HBC/HBG formulae of the previous section (no corrections to be added in this case, see §4.5).

In the following subsections, the formulae to compute the adjusted solution for the new unknowns and the corrections of the original ones for each case will be deduced. If the network does not have junction unknowns the possibilities are reduced to two: first case and fourth case. The corresponding solution is obtained by setting *B* and *D* to zero in these cases. Moreover steps 3 and 4 of the general routine introduced have no place in this situation but only steps 1, 2 and 5.

The original network has *n* blocks and its adjusted interior and junction vectors are *x*_0_ and *y*_0_, respectively. To this network, *N* new blocks are to be inserted in each case and the corrections to the interior and to the junction vector are d*x* and d*y*, respectively. For first and third case blocks *N*≤*n* as they are linked with the interior unknowns of the original network; if *N*<*n*, the blocks of unmatched unknowns are to be filled with corresponding null matrices instead of *C*_*i*_ and *D*_*i*_. For second and fourth case blocks *N* is boundless.

The original network is represented by a matrix *A* for the interior unknowns and a matrix *B* for the junction unknowns and in like manner, the blocks to be inserted are represented by a matrix of interior unknowns *C* and a matrix of junction unknowns *D*. The matrices *A*, *B*, *C* and *D* relate to the matrices for each block as follows:

$$\begin{array}{rl} A & =\left(\begin{array}{cccc} A_{1} & & & \\ & A_{2} & & \\ & & \ddots & \\ & & & A_{n} \end{array}\right)\;\text{and}\;B=\left(\begin{array}{c} B_{1}\\ B_{2}\\ \vdots \\ B_{n} \end{array}\right)\\ C & =\left(\begin{array}{cccc} C_{1} & & & \\ & C_{2} & & \\ & & \ddots & \\ & & & C_{N} \end{array}\right)\;\text{and}\;D=\left(\begin{array}{c} D_{1}\\ D_{2}\\ \vdots \\ D_{N} \end{array}\right). \end{array}$$

Moreover, the adjusted solution of the original network relates to its blocks and observations as follows:

4.1 $$\begin{array}{rl} \left(\begin{array}{cc} A & B \end{array}\right)\left(\begin{array}{c} x\\ y \end{array}\right)-l & =v\overset{v^{t}v\to \text{min}}{⟹}{\left(\begin{array}{cc} A & B \end{array}\right)}^{t}\left(\begin{array}{cc} A & B \end{array}\right)\left(\begin{array}{c} x\\ y \end{array}\right)\\ & ={\left(\begin{array}{cc} A & B \end{array}\right)}^{t}l⟹\left\{\begin{array}{l} A^{t}Ax_{0}+A^{t}By_{0}=A^{t}l\\ B^{t}Ax_{0}+B^{t}By_{0}=B^{t}l. \end{array}\right. \end{array}$$

After deducing the corresponding formulae for each case from equation (4.1) the formulae considering weights for the observations can be done as in §3.2. The ultimate formulae including weight matrices *P* and *Q* for the original set of observations and for the set of observations to be inserted, respectively, can be obtained by a change of variables. *P* is given by equation (3.20) and *Q* is likewise a diagonal block matrix, i.e. *Q*=diag(*Q*_1_,*Q*_2_,…,*Q*_*N*_).

### 4.2 First case blocks

In this case, the new blocks comprise observations for the unknowns of the original network only. The new equation of the network is as follows:

$$\left(\begin{array}{cc} A & B\\ C & D \end{array}\right)\left(\begin{array}{c} x_{0}+dx\\ y_{0}+dy \end{array}\right)-\left(\begin{array}{c} l\\ l_{p} \end{array}\right)=\left(\begin{array}{c} v+dv\\ v_{p} \end{array}\right).$$

Minimizing the sum of the squares of the new residuals, i.e. (*v*+d*v* *v*_*p*_)(*v*+d*v* *v*_*p*_)^*t*^, incurs into the following block system:

$$\begin{array}{rl} & \left(\begin{array}{cc} A^{t} & C^{t}\\ B^{t} & D^{t} \end{array}\right)\left(\begin{array}{cc} A & B\\ C & D \end{array}\right)\left(\begin{array}{c} x_{0}+dx\\ y_{0}+dy \end{array}\right)=\left(\begin{array}{cc} A^{t} & C^{t}\\ B^{t} & D^{t} \end{array}\right)\left(\begin{array}{c} l\\ l_{p} \end{array}\right)\\ & \overset{\text{equation (4.1)}}{⟹}\;\left\{\begin{array}{c} \left(A^{t}A+C^{t}C)\;dx+(A^{t}B+C^{t}D)\;dy=C^{t}(l_{p}-Cx_{0}-Dy_{0}\right)\\ \left(B^{t}A+D^{t}C)\;dx+(B^{t}B+D^{t}D)\;dy=D^{t}(l_{p}-Cx_{0}-Dy_{0}\right) \end{array}\right.\\ & \Rightarrow [A^{t}A+C^{t}C-(A^{t}B+C^{t}D){\left(B^{t}B+D^{t}D\right)}^{-1}(B^{t}A+D^{t}C)]\;dx\\ & =[C^{t}-(A^{t}B+C^{t}D){\left(B^{t}B+D^{t}D\right)}^{-1}D^{t}](l_{p}-Cx_{0}-Dy_{0})\\ \;\text{and}\;(B^{t}B+D^{t}D)\;dy & =D^{t}(l_{p}-Cx_{0}-Dy_{0})-(B^{t}A+D^{t}C)\;dx. \end{array}$$

In the light of the division into *n* and *N* blocks, the corrections are as follows:

$$\begin{array}{rl} & [A_{i}^{t}A_{i}+C_{i}^{t}C_{i}-(A_{i}^{t}B_{i}+C_{i}^{t}D_{i}){\left[\Sigma (B_{j}^{t}B_{j}+D_{j}^{t}D_{j})\right]}^{-1}(B_{i}^{t}A_{i}+D_{i}^{t}C_{i})]\;dx_{i}\\ & \;=[C_{i}^{t}-(A_{i}^{t}B_{i}+C_{i}^{t}D_{i}){\left[\Sigma (B_{j}^{t}B_{j}+D_{j}^{t}D_{j})\right]}^{-1}D_{i}^{t}](l_{pi}-C_{i}x_{0i}-D_{i}y_{0}),\;\forall i\leq n \end{array}$$

and

$$\Sigma (B_{i}^{t}B_{i}+D_{i}^{t}D_{i})\;dy=\Sigma [D_{i}^{t}(l_{pi}-C_{i}x_{0i}-D_{i}y_{0})-(B_{i}^{t}A_{i}+D_{i}^{t}C_{i})\;dx_{i}].$$

In the light of the weight matrices *P* and *Q*, the corrections are as follows:

4.2 $$\begin{array}{rl} & [A_{i}^{t}P_{i}A_{i}+C_{i}^{t}Q_{i}C_{i}-(A_{i}^{t}P_{i}B_{i}+C_{i}^{t}Q_{i}D_{i}){\left[\Sigma (B_{j}^{t}P_{j}B_{j}+D_{j}^{t}Q_{j}D_{j})\right]}^{-1}(B_{i}^{t}P_{i}A_{i}+D_{i}^{t}Q_{i}C_{i})]\;dx_{i}\\ & \;=[C_{i}^{t}Q_{i}-(A_{i}^{t}P_{i}B_{i}+C_{i}^{t}Q_{i}D_{i}){\left[\Sigma (B_{j}^{t}P_{j}B_{j}+D_{j}^{t}Q_{j}D_{j})\right]}^{-1}D_{i}^{t}Q_{i}](l_{pi}-C_{i}x_{0i}-D_{i}y_{0}),\;\forall i\leq n \end{array}$$

and

4.3 $$\Sigma (B_{i}^{t}P_{i}B_{i}+D_{i}^{t}Q_{i}D_{i})\;dy=\Sigma [D_{i}^{t}Q_{i}(l_{pi}-C_{i}x_{0i}-D_{i}y_{0})-(B_{i}^{t}P_{i}A_{i}+D_{i}^{t}Q_{i}C_{i})\;dx_{i}].$$

### 4.3 Second case blocks

In this case, the new blocks share junction unknowns but have their own interior unknowns. The new equation of the network is as follows:

$$\left(\begin{array}{ccc} A & B & \\ & D & C \end{array}\right)\left(\begin{array}{c} x_{0}+dx\\ y_{0}+dy\\ x_{p} \end{array}\right)-\left(\begin{array}{c} l\\ l_{p} \end{array}\right)=\left(\begin{array}{c} v+dv\\ v_{p} \end{array}\right).$$

Minimizing the sum of the squares of the new residuals, i.e. (*v*+d*v* *v*_*p*_)(*v*+d*v* *v*_*p*_)^*t*^, incurs into the following block system:

$$\begin{array}{rl} & \left(\begin{array}{cc} A^{t} & \\ B^{t} & D^{t}\\ & C^{t} \end{array}\right)\left(\begin{array}{ccc} A & B & \\ & D & C \end{array}\right)\left(\begin{array}{c} x_{0}+dx\\ y_{0}+dy\\ x_{p} \end{array}\right)=\left(\begin{array}{cc} A^{t} & \\ B^{t} & D^{t}\\ & C^{t} \end{array}\right)\left(\begin{array}{c} l\\ l_{p} \end{array}\right)\\ & \;\overset{\text{equation (4.1)}}{⟹}\;\left\{\begin{array}{l} A^{t}A\;dx+A^{t}B\;dy=0\\ B^{t}A\;dx+(B^{t}B+D^{t}D)\;dy+D^{t}Cx_{p}=D^{t}(l_{p}-Dy_{0})\\ C^{t}D\;dy+C^{t}Cx_{p}=C^{t}(l_{p}-Dy_{0}) \end{array}\right.\\ & \;\Rightarrow \;[B^{t}B-B^{t}A{\left(A^{t}A\right)}^{-1}A^{t}B+D^{t}D-D^{t}C{\left(C^{t}C\right)}^{-1}C^{t}D]\;dy\\ & \;=[D^{t}-D^{t}C{\left(C^{t}C\right)}^{-1}C^{t}](l_{p}-Dy_{0}) \end{array}$$

and

$$\left\{\begin{array}{l} C^{t}Cx_{p}=C^{t}(l_{p}-Dy_{0})-C^{t}D\;dy\\ -A^{t}A\;dx=A^{t}B\;dy. \end{array}\right.$$

In the light of the division into *n* and *N* blocks, the corrections are as follows:

$$\begin{array}{rl} & \Sigma [B_{i}^{t}B_{i}-B_{i}^{t}A_{i}{\left(A_{i}^{t}A_{i}\right)}^{-1}A_{i}^{t}B_{i}+D_{i}^{t}D_{i}-D_{i}^{t}C_{i}{\left(C_{i}^{t}C_{i}\right)}^{-1}C_{i}^{t}D_{i}]\;dy\\ & \;=\Sigma [D_{i}^{t}-D_{i}^{t}C_{i}{\left(C_{i}^{t}C_{i}\right)}^{-1}C_{i}^{t}](l_{pi}-D_{i}y_{0})\\ & \left\{\begin{array}{l} C_{i}^{t}C_{i}x_{\mathrm{pi}}=C_{i}^{t}(l_{pi}-D_{i}y_{0})-C_{i}^{t}D_{i}\;dy,\;\forall i\leq N\\ -A_{i}^{t}A_{i}\;dx_{i}=A_{i}^{t}B_{i}dy,\;\forall i\leq n. \end{array}\right. \end{array}$$

In the light of the weight matrices *P* and *Q*, the corrections are as follows:

4.4 $$\begin{array}{rl} & \Sigma [B_{i}^{t}P_{i}B_{i}-B_{i}^{t}P_{i}A_{i}{\left(A_{i}^{t}P_{i}A_{i}\right)}^{-1}A_{i}^{t}P_{i}B_{i}+D_{i}^{t}Q_{i}D_{i}-D_{i}^{t}Q_{i}C_{i}{\left(C_{i}^{t}Q_{i}C_{i}\right)}^{-1}C_{i}^{t}Q_{i}D_{i}]\;dy\\ & \;=\Sigma [D_{i}^{t}Q_{i}-D_{i}^{t}Q_{i}C_{i}{\left(C_{i}^{t}Q_{i}C_{i}\right)}^{-1}C_{i}^{t}Q_{i}](l_{pi}-D_{i}y_{0}) \end{array}$$

and

4.5 $$\left.\begin{array}{rl} C_{i}^{t}Q_{i}C_{i}x_{pi} & =C_{i}^{t}Q_{i}(l_{pi}-D_{i}y_{0})-C_{i}^{t}Q_{i}D_{i}\;dy,\;\forall i\leq N\\ A_{i}^{t}P_{i}A_{i}\;dx_{i} & =-A_{i}^{t}P_{i}B_{i}\;dy,\;\forall i\leq n. \end{array}\right\}$$

### 4.4 Third case blocks

In this case, the new blocks share interior unknowns but have their own junction unknowns. The new equation of the network is as follows:

$$\left(\begin{array}{ccc} A & B & \\ C & & D \end{array}\right)\left(\begin{array}{c} x_{0}+dx\\ y_{0}+dy\\ y_{p} \end{array}\right)-\left(\begin{array}{c} l\\ l_{p} \end{array}\right)=\left(\begin{array}{c} v+dv\\ v_{p} \end{array}\right).$$

Minimizing the sum of the squares of the new residuals, i.e. (*v*+d*v* *v*_*p*_)(*v*+d*v* *v*_*p*_)^*t*^, incurs into the following block system:

$$\begin{array}{rl} & \left(\begin{array}{cc} A^{t} & C^{t}\\ B^{t} & \\ & D^{t} \end{array}\right)\left(\begin{array}{ccc} A & B & \\ C & & D \end{array}\right)\left(\begin{array}{c} x_{0}+dx\\ y_{0}+dy\\ y_{p} \end{array}\right)=\left(\begin{array}{cc} A^{t} & C^{t}\\ B^{t} & \\ & D^{t} \end{array}\right)\left(\begin{array}{c} l\\ l_{p} \end{array}\right)\\ & \;\overset{\text{equation (4.1)}}{⟹}\;\left\{\begin{array}{l} \left(A^{t}A+C^{t}C)\;dx+A^{t}B\;dy+C^{t}Dy_{p}=C^{t}(l_{p}-Cx_{0}\right)\\ B^{t}A\;dx+B^{t}B\;dy=0\\ D^{t}C\;dx+D^{t}Dy_{p}=D^{t}(l_{p}-Cx_{0}) \end{array}\right.\\ & \;\Rightarrow \;D^{t}[D-C{\left(A^{t}A-A^{t}B{\left(B^{t}B\right)}^{-1}B^{t}A+C^{t}C\right)}^{-1}C^{t}D]y_{p}\\ & \;=[D^{t}-D^{t}C{\left(A^{t}A-A^{t}B{\left(B^{t}B\right)}^{-1}B^{t}A+C^{t}C\right)}^{-1}C^{t}](l_{p}-Cx_{0}) \end{array}$$

and

$$\begin{array}{r} \left\{\begin{array}{l} {}[A^{t}A-A^{t}B{\left(B^{t}B\right)}^{-1}B^{t}A+C^{t}C]\;dx=C^{t}(l_{p}-Cx_{0})-C^{t}Dy_{p}\\ -B^{t}B\;dy=B^{t}A\;dx. \end{array}\right. \end{array}$$

In the light of the division into *n* and *N* blocks, the corrections are as follows:

$$\begin{array}{rl} & \Sigma [D_{i}^{t}D_{i}-D_{i}^{t}C_{i}{\left(A_{i}^{t}A_{i}-A_{i}^{t}B_{i}{\left(B_{i}^{t}B_{i}\right)}^{-1}B_{i}^{t}A_{i}+C_{i}^{t}C_{i}\right)}^{-1}C_{i}^{t}D_{i}]y_{p}\\ & \;=\Sigma [D_{i}^{t}-D_{i}^{t}C_{i}{\left(A_{i}^{t}A_{i}-A_{i}^{t}B_{i}{\left(B_{i}^{t}B_{i}\right)}^{-1}B_{i}^{t}A_{i}+C_{i}^{t}C_{i}\right)}^{-1}C_{i}^{t}](l_{p_{i}}-C_{i}x_{0i})\\ & \left\{\begin{array}{l} {}[A_{i}^{t}A_{i}-A_{i}^{t}B_{i}{\left(B_{i}^{t}B_{i}\right)}^{-1}B_{i}^{t}A_{i}+C_{i}^{t}C_{i}]\;dx_{i}=C_{i}^{t}(l_{p_{i}}-C_{i}x_{0i})-C_{i}^{t}D_{i}y_{p},\;\forall i\leq n\\ \Sigma (B_{i}^{t}B_{i})\;dy=-\Sigma B_{i}^{t}A_{i}\;dx_{i}. \end{array}\right. \end{array}$$

In the light of the weight matrices *P* and *Q*, the corrections are as follows:

4.6 $$\begin{array}{rl} & \Sigma [D_{i}^{t}Q_{i}D_{i}-D_{i}^{t}Q_{i}C_{i}{\left(A_{i}^{t}P_{i}A_{i}-A_{i}^{t}P_{i}B_{i}{\left(B_{i}^{t}P_{i}B_{i}\right)}^{-1}B_{i}^{t}P_{i}A_{i}+C_{i}^{t}Q_{i}C_{i}\right)}^{-1}C_{i}^{t}Q_{i}D_{i}]y_{p}\\ & \;=\Sigma [D_{i}^{t}Q_{i}-D_{i}^{t}Q_{i}C_{i}(A_{i}^{t}P_{i}A_{i}-A_{i}^{t}P_{i}B_{i}{{\left(B_{i}^{t}P_{i}B_{i}\right)}^{-1}B_{i}^{t}P_{i}A_{i}+C_{i}^{t}Q_{i}C_{i})}^{-1}C_{i}^{t}Q_{i}](l_{p_{i}}-C_{i}x_{0i}) \end{array}$$

and

4.7 $$\left.\begin{array}{rl} & [A_{i}^{t}P_{i}A_{i}-A_{i}^{t}P_{i}B_{i}{\left(B_{i}^{t}P_{i}B_{i}\right)}^{-1}B_{i}^{t}P_{i}A_{i}+C_{i}^{t}Q_{i}C_{i}]\;dx_{i}=C_{i}^{t}Q_{i}(l_{p_{i}}-C_{i}x_{0i})-C_{i}^{t}Q_{i}D_{i}y_{p},\;\forall i\leq n\\ & \Sigma (B_{i}^{t}P_{i}B_{i})\;dy=-\Sigma B_{i}^{t}P_{i}A_{i}\;dx_{i}. \end{array}\right\}$$

### 4.5 Fourth case blocks

In this case, the new blocks have no share in interior unknowns nor in junction unknowns. The new equation of the network is as follows:

$$\left(\begin{array}{cccc} A & B & & \\ & & C & D \end{array}\right)\left(\begin{array}{c} x_{0}+dx\\ y_{0}+dy\\ x_{p}\\ y_{p} \end{array}\right)-\left(\begin{array}{c} l\\ l_{p} \end{array}\right)=\left(\begin{array}{c} v+dv\\ v_{p} \end{array}\right).$$

Letting (*A* *B*)=*E*, (*C* *D*)=*F*, (*x*_0_ *y*_0_)^*t*^=*z*_0_ and (*x*_*p*_ *y*_*p*_)^*t*^=*z*_*p*_ and minimizing the sum of the squares of the new residuals, i.e. (*v*+d*v* *v*_*p*_)(*v*+d*v* *v*_*p*_)^*t*^, incurs into the following block system:

$$\left(\begin{array}{cc} E^{t} & \\ & F^{t} \end{array}\right)\left(\begin{array}{cc} E & \\ & F \end{array}\right)\left(\begin{array}{c} z_{0}+dz\\ z_{p} \end{array}\right)=\left(\begin{array}{cc} E^{t} & \\ & F^{t} \end{array}\right)\left(\begin{array}{c} l\\ l_{p} \end{array}\right)\overset{\text{equation (4.1)}}{⟹}\left\{\begin{array}{l} E^{t}E\;dz=0\\ F^{t}Fz_{p}=F^{t}l_{p}, \end{array}\right.$$

which implies d*z*=0 and thus that the adjustment of the new blocks is to be done separately as it has no influence in the original adjusted solution. The adjustment of the new blocks in this case can be done using the HB formulae deduced in the previous section.

## 5. Implementation

### 5.1 Background

Though the number of observations was very large in [9], about six million, the unknowns were small in amount. So, Lema *et al.*'s [9] simulated levelling network is far from a real one for two reasons: unknowns vastly outnumbered by observations and too few junction unknowns—only 9. Such a small junction vector disables an evaluation of the numerical performance of the proposed HBC and HBG techniques by means of efficiency. Larger junction unknowns vectors are required to sense the difference in the elapsed CPU times taken by HBC and HBG. Concurrently, large continental geodetic networks involve hundreds of thousands of variables and the number of observations is much closer to the actual amount of unknowns to be determined. Moreover, the junction unknowns usually have a comparable size to that of the interior unknowns. And the same holds for the number of blocks and subvectors [2].

Here, we have generated levelling networks of higher dimensions and also levelling networks with a much weightier junction vector (usually about 2/3 of the number of interior unknowns) with observations that slightly outnumber the unknowns. This way, the generated levelling networks are much more realistic and the results thus obtained are much more significant and descriptive in terms of uncertainty and numerical efficiency. But linear systems with these characteristics demand much more memory to be allocated. This ended up constraining the actual size of such realistic geodetic networks that we were to build. So, these simulated networks we have built are a realistic but scaled continental geodetic network.

As it turned out from the final results, this scaled continental network was enough to evaluate the performance of the proposed techniques. The simulation highlighted the improvement provided by the HBC technique, especially in its numerical efficiency, as it outran HBG being about 33% faster. This was no surprise as predicted from the number of flops done by HBC and HBG, a fact pointed out in §3.1. Moreover, the solutions are precise to the extent that the absolute value of the difference between an unknown determined by HBC and HBG techniques never exceeded 10^−16^. In practice, they are numerically identical. However, when it comes to the elapsed times, then the differences arise.

Both here and in Lema *et al*. [9], we used personal computers with not more than 8 Gb of RAM. Therefore, we do not consider parallel programming even though our PCs have shared memory architecture. For realistic geodetic networks of continental sizes, it seems appropriate to consider the use of clusters in a distributed memory architecture so that much more memory and processors are available and parallelization of our proposed routines may significantly improve performance of the software tools.

If parallelization of HBC shows to be elusive or does not provide significant increase in efficiency a possible route is to consider other approaches to solve the block system that may be parallelized and take advantage of the distributed architecture. Then, these parallel alternatives to HBC should be compared with non-parallel HBC to check which is the most efficient.

### 5.2 The techniques and the levelling network

The solution given by the HB technique was determined using equations (3.18) and (3.9). To implement them we chose Fortran due to its numerical stability which, in conjunction with its useful matrix notation and derived data types, makes it helpful to store and to manipulate the blocks of the global geodetic network given by equation (3.18).

Basically, once the network was generated the solution takes place in two steps: first, the block system of equation (3.18) is solved by HBC or HBG technique; second, the junction solution vector obtained is substituted into equation (3.9) which returns the interior solution vector immediately. Note that the second step is identical for both techniques.

Before the first step is taken, the levelling network must be generated. The details can be found in appendix B.

### 5.3 The numerical tests

For each proposed technique, two attributes are evaluated: uncertainty and efficiency. To estimate the uncertainty, we calculated the maximum absolute difference between HBG and HBC solutions, after [13], as follows:

5.1 $$\Delta =\max|X_{\mathrm{HBC}}-X_{\mathrm{HBG}}|,$$

where *X*_HBC_ and *X*_HBG_ are the global adjusted solutions of the levelling network obtained by HBC and HBG, respectively. This way, the uncertainty of HBC which is the novel method can be evaluated by taking HBG as the reference because Wolf's [1] approach is very much like it.

The uncertainty defined this way is just a relative evaluation and asserts the agreement between the distinct solutions provided by each technique. The numerical uncertainty measured in this way is less than 10^−16^ using extended double precision (16 bytes) and 10^−13^ using double precision (8 bytes). Usually, given the large number of variables and thus of flops to be performed, single precision (4 bytes) is not suited for the adjustment of large continental geodetic networks due to the accumulation of round-off errors [2].

**Figure 2.**
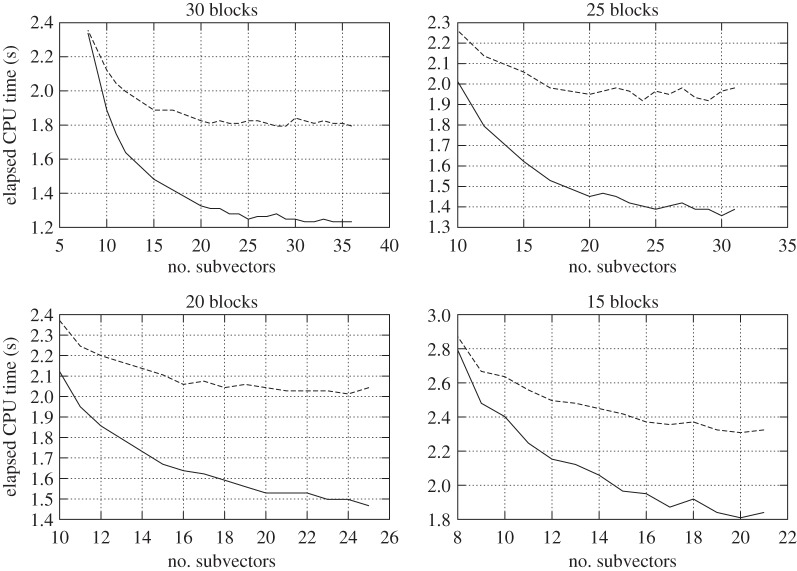
Elapsed CPU times to adjust the levelling network by HBC (solid line) and HBG (dashed line). The elapsed times are measured for each number of subvectors while the number of blocks is kept constant and the number of unknowns and observations varies smoothly as their final amount is randomly determined. The network comprises about 1800 interior unknowns and 1200 junction unknowns, with about 3200 observations. The weight matrix *P* was taken as the identity matrix and extended double precision was used to store floating-point numbers.

**Figure 3.**
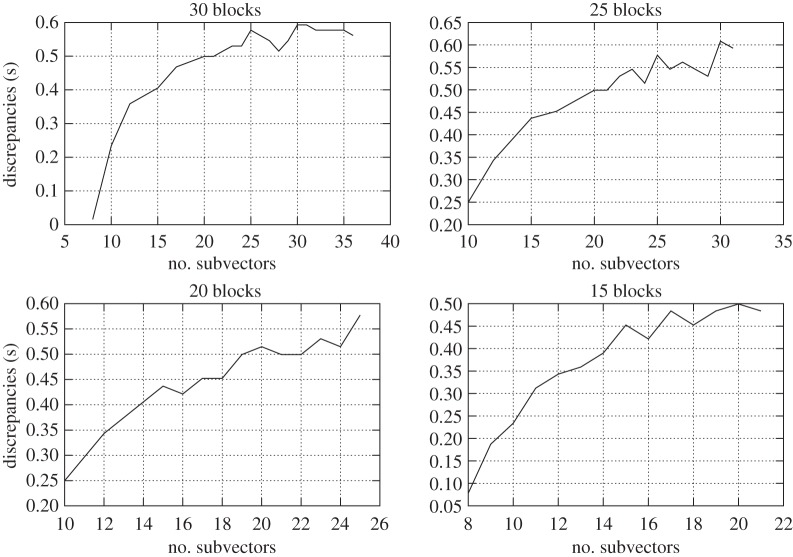
Discrepancies between the elapsed CPU times to adjust the levelling network by HBG and HBC. The same considerations as for figure 2 apply here.

To evaluate the numerical efficiency, we computed the elapsed CPU times to solve the block system in the junction unknowns and the back substitution to calculate the interior unknowns. The latter will always take a negligible time compared to that taken to solve the block system in the junction unknowns, which indicates that the approach to solve the junction vector is the key to the global performance of any HB-derived technique. Furthermore, for this set of measurements, given a number of blocks, the number of subvectors that yielded the least elapsed time was about the same as the number of blocks. The optimal efficiency did not deviate too much from this value. The results are displayed in figures 2 and 3.

From the resulting dataset, we computed statistics to determine the average and the maximum increase in efficiency provided by HBC as 100(*t*_HBG_−*t*_HBC_)/*t*_HBG_, where *t*_HBG_ and *t*_HBC_ are the elapsed CPU times taken by HBG and HBC to solve the levelling network under consideration. To determine these parameters, we set 2*σ* as a threshold to remove outliers as it provides a more likely confidence interval than the usual 1*σ* adopted in geodesy. Nevertheless, there was never more than one outlier in each case. These statistics are displayed in table 1. Furthermore, as the number of subvectors approached 0, the block system became closer to a typical linear system and the efficiency of both techniques converged to a similar elapsed time.

**Table 1.** Maximum and average increase in efficiency by means of elapsed CPU time provided by HBC technique against HBG technique. Standard deviation (s.d.) is also provided.

no. blocks	max. increase (%)	avg. increase (%)	s.d. (%)
30	33	28	5.9
25	31	27	3.7
20	29	22	4.1
15	22	17	4.7

## 6. Concluding remarks

The HB technique is an optimization of the LS method for the adjustment of large geodetic networks. The equivalence between HB and LS solutions was demonstrated through the deduction as HB was deduced from LS in its simplest formulation and properties of block matrices, as opposed to [1].

Fortran's usefulness derived simple yet robust codes for the softwares we have conceived. Its matrix notation and derived data types were adequate to design codes for the proposed HBC and HBG approaches as well. Wolf's [1] presented solution has hidden steps, as it is very concise and considers an approach very similar to HBG, but of poorer quality. Moreover, Wolf [1] considered different relations to derive the formulae which made the deduction unnecessarily less straightforward. Here, the HB technique is derived from the well-known LS in its simplest form and shown to be a particular case of it that takes place for the block-angular matrix of geodetic networks. Therefore, HBC and HBG are optimizations to the HB technique for large geodetic networks in which the junction vector has to be divided into subvectors.

The formulae to insert observations in a geodetic network whose solution is already known are also included in the paper. These are an alternate to readjusting the whole new network without inputing the solution computed for the original network. Instead of solving a linear system of increased size (original network unknowns plus new block unknowns), they provide a means to obtain the adjusted solution for the whole new network by solving linear systems with the number of unknowns equal to the number of unknowns being inserted and to the number of unknowns of the original network. This is a more efficient formulation that leads to linear systems of reduced size.

Here we showed that the HBC approach is about 33% faster than HBG and that when the junction unknowns vector is too large, a similar problem to that witnessed during previous work to allocate memory occurred in Fortran, which forced its division into subvectors as the networks' geometry conformed into realistic ones. In addition to these measurements of numerical efficiency, we measured the relative uncertainty between HBC and HBG solutions which never surpasses 10^−13^ under double precision. Therefore, HBC is more efficient and its use is recommended over HBG.

Moreover, the elapsed times to solve the junction system and the interior system highlighted that the approach to solve the junction unknowns block system is the key to achieve an optimal performance outweighing the approach to determine interior unknowns. Furthermore, in realistic cases it seems appropriate to consider computers with a distributed memory architecture that provide more memory and processing power. This way, we recommend the investigation of HBC's parallelization or of parallel alternatives to it. A survey of parallel routines for linear systems can be found in [14]. These might provide additional increases in efficiency.

Note that there is another general scheme to obtain an LS solution under HB. Here, we studied the approach by normal equations, which, under HB environment, have not witnessed so far the use of a block Choleski decomposition to solve the junction vector as proposed here. As opposed to normal equations, the literature presents the QR approach (i.e. to factorize a matrix into the product of an orthogonal matrix *Q* by an upper triangular matrix *R*) to obtain an LS solution, which gives two benchmarks to determine an LS solution: normal equations and QR factorization.

We have not covered QR factorization, nor is it covered in [1–6]. To the reader interested in further details about it we recommend [10], which reports an extensive study on how to use a block QR factorization to adjust a geodetic network under HB environment. And, as [11] points out, normal equations (the standard in geodesy) are usually more efficient, whereas QR factorization is usually more stable. Therefore, the choice of which approach to take in order to determine an LS solution for large geodetic networks merits a separate investigation. Moreover, there is not in the literature yet the consideration of other decomposition methods like the singular-value decomposition.

Moreover, with geodetic networks geometrically similar to the ones we have presented here but of larger scales, an evaluation of HBC normal equations against Golub & Plemmons's [10] block QR factorization would be useful.

## Data accessibility

Data have been deposited in Dryad, see .

## Appendix A. Implementation of HBC and HBG approaches

To implement HBC and HBG, we extended Del Rio & Oliveira's [15] Choleski and Gaussian elimination algorithms for linear systems by adapting them to solve a block system (these algorithms are widely known and can be found in other sources, e.g. Press *et al.*'s [12] algorithms). Concurrently, we used block dot product Cholesky [11] to factorize the system's block matrix into a product of lower and upper block triangular matrices. In §5, we show numerical tests in which the performance of an HBC is compared with that of an HBG.

## Appendix B. Generation of the block matrices for the simulation

As for this simulation, we start with a pair of seed matrices *a* and *b* from which the matrices *A*_*i*_ and *B*_*i*_ of each block *i* are to be generated. These matrices are very sparse and the non-null elements are either −1 or 1, since this is a levelling network. So, to form the seed matrices, an iteration runs first at the rows and then at the columns performing the same action: to randomly choose an element and to randomly assign it the value 1 or −1, while leaving the others null. This way, we avoid singularity by ensuring that there are no null-rows nor null-columns. But, singularity can still occur if there are two identical rows or two identical columns. Therefore, to avoid this kind of singularity as well, the entire actions described thus far are repeated 30 times as another iteration. As for the observations vector, 13 values are manually chosen and recorded in an input txt file (these observations were taken from block 1's in figure 1). From them, the remaining values are obtained by adding a random number between −20 and 20 to one of these 13 numbers, the one of these also chosen randomly until the *a*'s number of rows is matched. This ends up forming a seed *l* for each block's observations vector.

Once the seed matrices are generated, the matrices of each block and the submatrices and subvectors are formed by randomly picking a line from matrix *a* as for the *A*_*i*_ matrices and an element from vector *l* as for the *L*_*i*_ vectors and by randomly picking a column as for the *B*_*i*_ submatrices (the number of inner unknowns at each block is also randomly determined, so that their distribution over the blocks is inhomogeneous). Actually, the matrices *B*_*i*_ are not formed but only their submatrices. Therefore, the matrices *B*_*di*_ are formed by randomly picking a column from seed matrix *b*. The remaining submatrices that form the block system in the junction unknowns subvector are obtained from equations (3.12)–(3.14), (3.16) and (3.17) to yield the block system given by equation (3.18).

## References

[1] Wolf H 1978 The Helmert Block method—its origin and development. In Annals of the 2nd Int. Symp. on Problems Related to the Redefinition of North American Geodetic Networks Washington, DC: US Department of Commerce.

[2] Schwarz CR, Wade EB 1990 The North American datum of 1983: project methodology and execution. Bull. Geodesique 64, 28–62. (doi:10.1007/BF02530614)

[3] Costa SMA, Fortes LPS 1993 Resultados Preliminares do Ajustamento da Rede Planimétrica do Sistema Geodésico Brasileiro. Rio de Janeiro, Brazil: IBGE.

[4] Costa SMA, Fortes LPS 1991 Ajustamento da Rede Planimétrica do Sistema Geodésico Brasileiro. Rio de Janeiro, Brazil: IBGE.

[5] Pina WH, Pinheiro R, Santos CC, Pereira NR, Goldani D 2007 Reajustamento Global da Rede Altimétrica de Alta Precis ao do Brasil–RAAP. Rio de Janeiro, Brazil: IBGE.

[6] Pinheiro R, Goldani D, Santos CC, Pereira NR, Pina WH 2007 Situaç ao Atual do Ajustamento da Rede Altimétrica de Alta Precis ao–RAAP do SGB, Utilizando Sistema GHOST. Rio de Janeiro, Brazil: IBGE.

[7] Panafidina N, Malkin Z, Weber R 2006 A new combined European permanent network station coordinates solution. J. Geod. 80, 373–380. (doi:10.1007/s00190-006-0076-2)

[8] Nocquet J-M, Willis P, Garcia S 2006 Plate kinematics of Nubia Somalia using a combined DORIS and GPS solution. J. Geod. 80, 591–607. (doi:10.1007/s00190-006-0078-0)

[9] Lema ES, Cavassa CE, Oliveira L 2010 A Técnica de Helmert-blocking: Deduç ao e Implementaç ao em Redes Altimétricas Simuladas. In Proc. III Simpósio Brasileiro de Ciências Geodésicas e Tecnologias da Geoinformaç ao, Recife, PE, Brazil.

[10] Golub G, Plemmons R 1980 Large-scale geodetic least-squares adjustment by dissection and orthogonal decomposition. Linear Algebra Appl. 34, 3–27. (doi:10.1016/0024-3795(80)90156-1)

[11] Golub G, VanLoan CF 1996 Matrix computations. Baltimore, MD: The Johns Hopkins University Press.

[12] Press WH, Teukolsky SA, Vetterling WT, Flannery BP 2007 Numerical recipes, 3rd edn Cambridge, UK: Cambridge University Press.

[13] Del Rio E, Ferreira LF 2013 An expression of uncertainty and its application to positioning: a quality-metric and optimal ranges for the identification of cells with RFID. See https://www.mendeley.com/download/public/12369301/5616483454/0b84e39dc09dc93e5c45ec87bd84fe97690d2325/dl.pdf. 10.1186/s40064-015-1084-6 PMC4513044 26217551

[14] Heath M 2014 Parallel numerical algorithms. Lecture notes See https://courses.engr.illinois.edu/cs554/fa2013/notes/index.html (accessed 1 November 2014).

[15] Dieguez JPP 2005 Métodos de Cálculo Numérico. Fundaç ao Ricardo Franco. Rio de Janeiro, Brazil: Instituto Militar de Engenharia Coleção Disseminar.

